# Microsurgical resection of tumors of the lateral and third ventricles: operative corridors for difficult-to-reach lesions

**DOI:** 10.1007/s11060-016-2126-9

**Published:** 2016-05-27

**Authors:** Ulas Cikla, Kyle I. Swanson, Abdulfettah Tumturk, Nese Keser, Kutluay Uluc, Aaron Cohen-Gadol, Mustafa K. Baskaya

**Affiliations:** 1 Department of Neurological Surgery, School of Medicine, University of Wisconsin-Madison, CSC, K4/822, 600 Highland Avenue, Madison, WI 53792 USA; 2 Goodman Campbell Brain and Spine, Indiana University Department of Neurological Surgery, Indianapolis, IN USA

**Keywords:** Lateral ventricle, Third ventricle, Surgical approach, Microneurosurgery, Brain tumor surgery

## Abstract

Tumors of the lateral and third ventricles are cradled on all sides by vital vascular and eloquent neural structures. Microsurgical resection, which always requires attentive planning, plays a critical role in the contemporary management of these lesions. This article provides an overview of the open microsurgical approaches to the region highlighting key clinical perspectives.

**Electronic supplementary material:**

The online version of this article (doi:10.1007/s11060-016-2126-9) contains supplementary material, which is available to authorized users.

## Introduction

The surgical management of tumors of the lateral ventricles (LV) and the third ventricle (TV) remains a distinct challenge for neurosurgeons due to the deep and difficult-to-reach location and frequent involvement of adjacent critical neurovascular structures. An appropriate surgical approach should provide adequate operative working space with minimal brain retraction or brain transgression [1–3]. To accomplish these goals, neurosurgeons may choose an approach that necessitates a longer distance to reach the tumor if it minimizes the amount of brain tissue that is resected or placed at risk by the approach. Furthermore, selection of the optimal approach to ventricular tumors depends on multiple other factors including the size of the ventricles and the tumor, the location of the arterial supply, pathological features of the tumor, and the surgeon’s experience. This paper provides an overview of the open surgical operative corridors to the lateral and TV tumors, highlighting the key surgical principles.

## Lateral ventricles

The LV are anatomically divided into five parts: the body, atrium, frontal horn, temporal horn, and occipital horn [4]. Tumors of the LV can also be grouped into primary and secondary tumors. Primary tumors are those arising from the structures within the ventricle, whereas secondary tumors are the larger group of tumors derived from adjacent structures and expanding into the ventricular cavity. Overall, tumors of the LV comprise between 0.8 and 1.6 % of all brain tumors [5, 6]. As many of the tumors arising in the LV are benign and slow growing, they are often not detected until they reach a considerable size that causes obstructive hydrocephalus or mass effect. Headaches and visual changes, often related to hydrocephalus, are the most common presenting symptoms. Other symptoms include endocrine disturbance, motor and sensory deficits, nausea and vomiting, and cognitive impairment [5, 7–9].

**Fig. 1:**
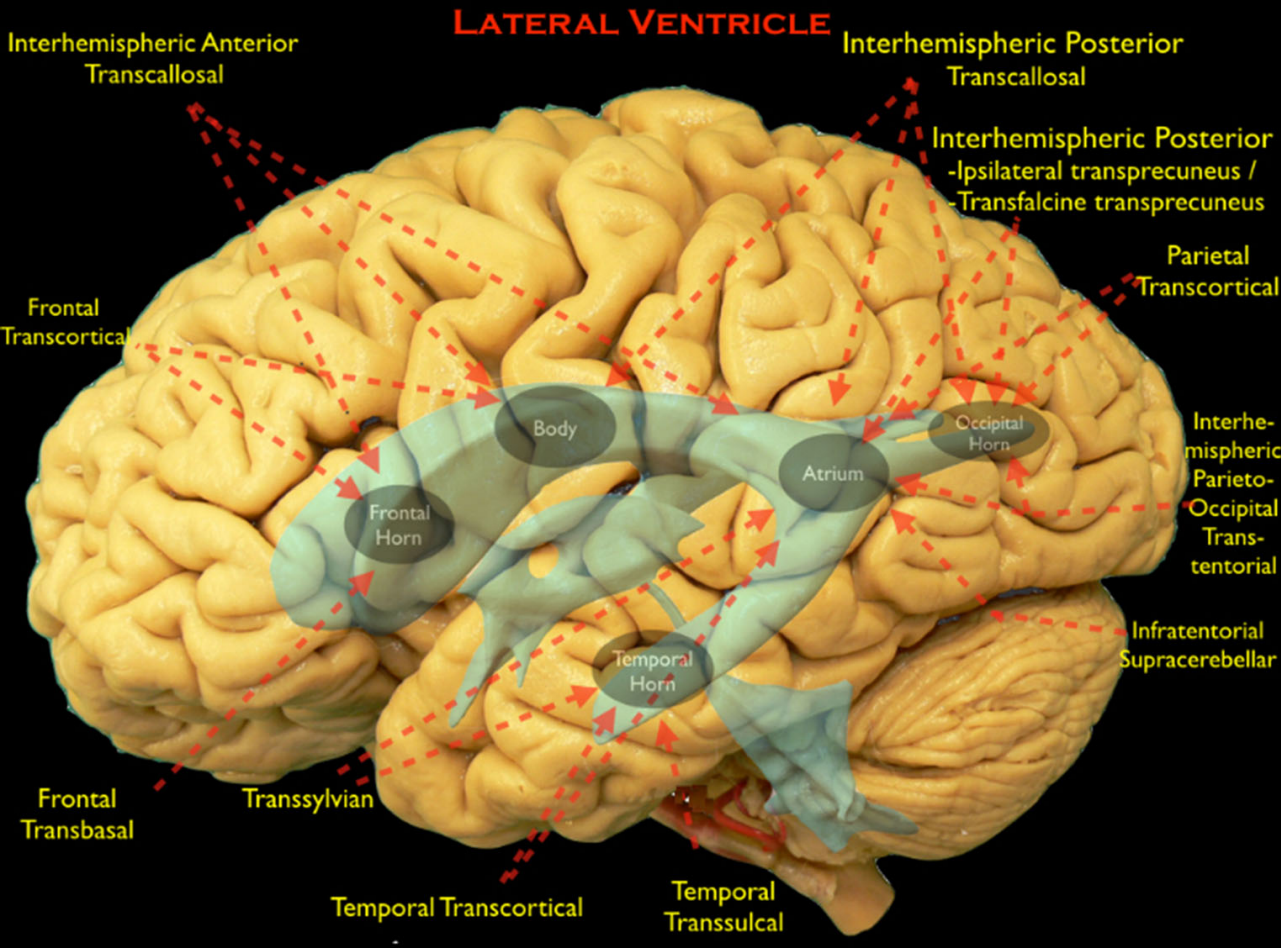
The surgical approaches to the lateral ventricle (LV) are shown on a lateral view of a cadaveric dissection of the brain. LV and third ventricle (TV) are shown in *blue*. Anatomical portions of the LV are depicted with *gray ellipses*. *Red arrows* show the direction of the approaches and the parts of the LV that can be reached by that individual approach

Multiple surgical approaches have been described for each location in the LV system (Fig. 1). The aim of each of these approaches is to provide an adequate corridor to the tumor while preserving eloquent overlying neurovascular structures [5]. A careful review of the pathoanatomy from multiple planes on imaging studies, including MRI, MR angiography or venography, and occasionally digital subtraction angiography, is essential for selecting the appropriate surgical strategy [10–12]. The neurovascular anatomy may be distorted by the tumor, or the patient may have an anatomical variation that makes a particular route unsafe. A thorough knowledge of the anatomy and available alternative surgical routes allows the neurosurgeon to accommodate to any change in the operative agenda and provides alternative contingency plans to deal with any unforeseen difficulty.

## Open surgical approaches to LV

### Frontal horn and body of LV

**Fig. 2:**
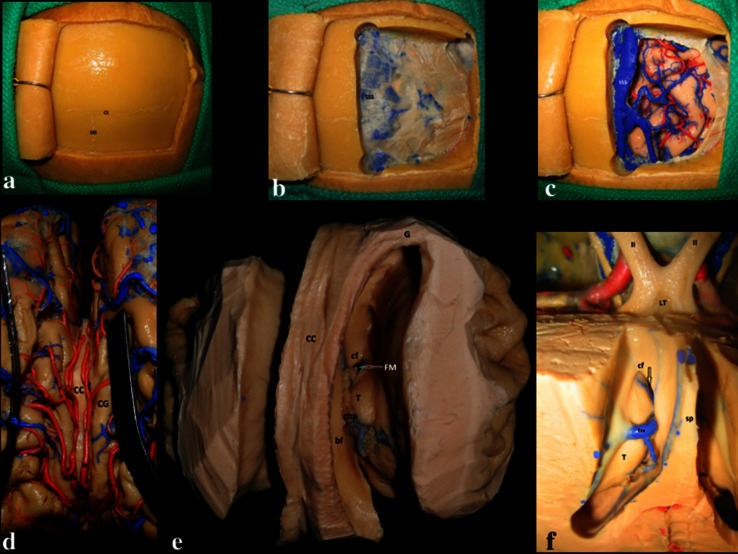
**a**–**f** Cadaveric dissection demonstrating the steps of interhemispheric transcallosal approach. **a** A C-shaped incision for the interhemispheric approach. **b** After craniotomy, the superior sagittal sinus (SSS) is seen at the midline. **c** After elevation of the dura, vasculature of the region, including cortical veins draining into the SSS, is seen more clearly. **d** The corpus callosum (CC), cingulate gyrus (CG), and the pericallosal arteries are seen in the interhemispheric fissure. **e** Dissection demonstrating the anatomical relationships of the LVs. Corpus callosum (CC), column of fornix (cf), foramen of Monro (FM), thalamus (T), genu of CC (G), body of fornix (bf), choroid plexus (chp). **f** Superior view of the LV over the FM (arrow) demonstrating the close relationship of the third ventricle with the optic nerve and the lamina terminalis. Optic nerve (II), septum pellucidum (sp), choroid plexus (chp), thalamus (T), thalamostriate vein (tsv), lamina terminalis (LT), column of the fornix (CF)

Tumors in and around the anterior two-thirds of the LV can be accessed via either the interhemispheric anterior transcallosal approach (IATcA) (Fig. 2) or the frontal transcortical approach (FTA) [13–15]. AITcA and FTA both allow for excellent visualization of LV anatomical landmarks, including the thalamostriatal, anterior-septal and caudate veins, foramen of Monro and choroid plexus (CP) [4].

FTA may provide better access to larger tumors than the IATcA in the frontal horn, but it has a limited exposure of the contralateral LV and may pose an increased risk of postoperative seizures [1, 5]. FTA requires transection of the cortex and therefore potentially carries a higher risk of postoperative neurologic decline, such as attention deficits, as compared to the limited callosotomy during the IATcA. A corticotomy in the middle frontal gyrus or dissection through the superior frontal sulcus well anterior to the motor cortex decreases the likelihood of significant neurological deficit, but either a corticotomy or retraction of the supplemental motor or premotor area may cause at least a temporary hemiparesis. Furthermore, FTA is usually not advised for tumors within the mid-body of the LV because this approach would require an extension of the cortical incision into the motor cortex [13]. The most frequent complications following FTA are epilepsy (26 % of patients) followed by transient mutism (11 % of patients), hemiparesis (7 % of patients), and short-term memory disturbance [15].

AITcA remains the most commonly preferred microsurgical approach for exposure of ventricular tumors. The head is often positioned so the superior sagittal sinus (SSS) is parallel to the floor, exploiting gravity retraction on the ipsilateral hemisphere away from the falx and SSS. Some colleagues position the head in a neutral position to maintain basic anatomical orientation during microsurgery. A horseshoe or a linear parasagittal skin incision allows a parasagittal craniotomy located two-thirds anterior and one-third posterior to the coronal suture guided by intraoperative image-based neuronavigation.

**Fig. 3:**
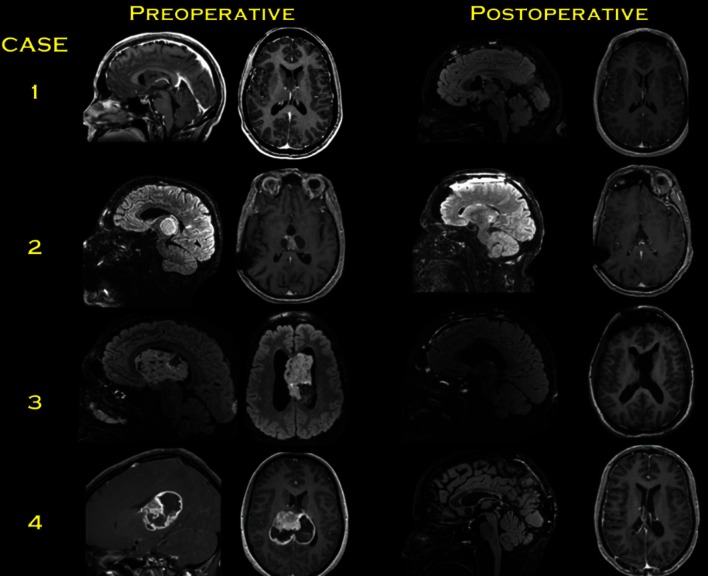
Preoperative and postoperative MR images of the cases which are presented in the complementary videos of the article. *Case 1* Sagittal and axial MRI with contrast show non-enhancing right LV tumor. Post-operative sagittal flair imaging shows the minimal callosotomy and axial post-contrast T1 imaging confirms gross total resection through interhemispheric transcallosal approach. [Please see the video 1]. *Case 2* Sagittal flair MRI and axial post-contrast T1 MRI shows a heterogeneously enhancing cystic tumor in the posterior TV. Post-operative sagittal cube MRI and post-contrast axial T1 MRI confirms gross total removal through this approach. [Please see the video 2]. *Case 3* Sagittal and axial flair MRI show a tumor occupying the frontal horn, body and atrium of the LV. Post-operative sagittal flair MRI show the extent of the callosotomy and axial T1 MRI confirms the gross total removal. [Please see the video 3]. *Case 4* The extent of heterogeneously enhancing tumor originating from the thalamus and the peripheral edema due to mass effect are shown in the contrasted sagittal and axial MRI. Postoperative sagittal flair MRI and post-contrast axial MRI confirm the gross total resection via posterior interhemispheric approach. [Please see the video 4]

The craniotomy is usually eccentric to one side but extends across the midline to allow for gentle mobilization of the SSS and falx cerebri. The dura is opened in a semilunar fashion with the SSS serving as the base of the dural flap. The dural incisions are tailored according to the drainage pattern of the parasagittal bridging veins. Every effort should be made to preserve the cortical draining veins and minimize the risk of venous infarction. Next, the interhemispheric fissure is dissected using meticulous sharp arachnoid dissection to free the cortex of the medial surface of the superior frontal gyrus from the falx cerebri. At the depth of the interhemispheric fissure, the corpus callosum (CC) is encountered and is differentiated from the cingulate gyri by a pearly white appearance. The cingulate gyri can be very adherent, requiring operator’s patience and adherence to microsurgical principles for their separation. The pericallosal arteries (PeCas) coursing over the CC are identified and carefully separated. Classically, the callosotomy involves an incision no larger than 2 cm, located in the midline between the two PeCas [16]. The exact location of the callosotomy can also be determined by neuronavigation. The target LV is entered after the callosotomy and anatomic landmarks are used to ensure that the correct LV has been entered. The surgical technique of the AITcA is demonstrated in videos presenting the resection of a LV subependymoma [Movie 1] and the resection of a LV gangliocytoma [Movie 2] (Fig. 3).

During the interhemispheric dissection, the cortices of the superior frontal and cingulate gyri, as well as the PeCas and their branches, are at risk of injury. Other potential major complications of this approach include disconnection syndrome from the callosotomy and transient or permanent memory deficits from injury to the forniceal bodies [3, 4, 13].

### Atrium of the LV

Multiple surgical corridors have been described to approach the atrium of the LV via various operative trajectories [17]. The interhemispheric posterior transcallosal approach (IPTcA) is preferred for lesions involving the atrium of the LV and the splenium of the CC. The surgical technique for this approach is demonstrated in a video of the resection of a grade IV astrocytoma involving the thalamus, both the LV and the splenium of the CC [Movie 3] (Fig. 3).

Yasargil described another key route to the ventricular trigone, the ipsilateral interhemispheric posterior parietooccipital approach (IPPoA) [7, 13, 14]. Lesions of the medial wall of the ventricular trigone and the TV posterior to the massa intermedia of the thalamus can be tackled by this approach [18]. Although this approach requires transection of a small area of the precuneus gyrus, it provides a safe route that minimizes the risk of injury to the optic radiations and visual cortex [14].

Izci et al. studied the microsurgical anatomy and topographical relation of the surgical corridor provided by the supracerebellar transtentorial transcollateral sulcus approach to the atrium [17]. This approach provides a long working distance to reach tumors located in the inferior part of the atrium and posterior parahippocampal gyrus; however, tumors with a notable extension above the tentorium, significant lateral extension or tumors growing into the TV are not usually amenable to this approach.

The transcortical approaches to the atrium risk traversing important white matter tracts such as the internal capsule, optic radiations, and the striate cortex [7]. The parietal transcortical approach (also called the superior parietal lobule (SPL) approach) is a traditional transcortical approach for access to both medial and lateral walls of the atrium by traversing less eloquent cortex [1, 12, 19–24]. After a cortical incision through the SPL, the atrium, posterior body of LV, posterior half of the TV, and the quadrigeminal cistern can be reached [4]. Of note, this route is usually employed when there is ventricular enlargement [13]. One potential disadvantage of the SPL approach is the inability to gain early control of the vascular supply to the tumor, which usually enters into the inferior aspect of the tumor [25]. The most common complication of this approach is a homonymous visual field deficit from injury to the optic radiations [26]. Injury to the adjacent eloquent dominant inferior parietal lobule, which includes the supramarginal and angular gyri, can result in Gerstmann syndrome (apraxia, acalculia, finger agnosia, and right-left confusion) [7, 12, 27]. To avoid these complications, the relation of the tumor to eloquent cortex should be carefully delineated on preoperative imaging and both anatomic landmarks and neuronavigation utilized intraoperatively to ensure protection of eloquent cortices.

The subtemporal approach is a very useful lateral route for removal of tumors localized in the atrium since this approach provides immediate access to the anterior choroidal artery, which often gives vascular supply to the tumor, and has a decreased incidence of visual field defects as compared to the transtemporal approach [28]. This approach is preferable when the ipsilateral temporal horn is large, and the tumor is relatively small. In larger tumors, the subtemporal approach may require excessive retraction on the temporal lobe to complete tumor resection [25]. Kawashima et al. demonstrated the efficacy of the subtemporal approach in which an incision is made in the inferior temporal gyrus, occipitotemporal gyrus, or collateral sulcus to avoid transgression of the optic radiations and speech centers located in the dominant hemisphere [18].

A less commonly used approach is the transtemporal approach, which utilizes a cortical incision through a portion of the middle or inferior temporal gyri [4, 7, 20–22, 26, 29, 30]. This approach risks homonymous quadrantanopia due to injury to the optic radiations, as well as limited or impaired recognition of emotions from injury to the non-dominant temporal lobe or receptive aphasia from injury to the dominant temporal lobe [31, 32].

### Temporal horn of LV

The temporal horn can be accessed via lateral transcortical trajectories, also called the transtemporal approaches, through the middle temporal gyrus, and less commonly the inferior temporal gyrus [7]. The transtemporal approach often provides the shortest trajectory to the lesions in the temporal horn and is greatly facilitated by dilated ventricles [13]. The transtemporal approach usually affords early access to the choroidal arterial pedicle, which is often the vascular supply of tumors in the temporal horn; early occlusion of these vascular feeders facilitates debulking of the tumor [33]. The inferior temporal gyrus route, though not as direct as the middle temporal gyrus route, can be used to provide a safe distance from the language area of the dominant temporal lobe and also to avoid the anterior fibers of the optic radiations. Care must be taken to prevent injury to the vein of Labbe, the primary drainage system of the lateral temporal lobe. The transtemporal approach can result in a partial upper-quadrantanopia though patients do not often perceive this deficit in daily activities [34]. Furthermore, choroidal artery territory infarcts can occur if the anterior choroidal artery is sacrificed while interrupting the vascular supply of the tumor [13].

For anterior temporal horn tumors, we advocate the transsylvian trajectory via the pterional approach as originally described by Yasargil. This approach allows entrance into the anterior temporal horn while minimizing the risk to the anterior loop of Meyer’s optic radiation fibers as long as rigid retraction is not applied to the temporal lobe [13, 14, 35, 36]. This approach requires a wide opening of the Sylvian fissure, which is technically more demanding than the transtemporal approach. The former also harbors the potential for injury to the arterial branches of the middle cerebral artery and the sylvian veins.

### Occipital horn of LV

For tumors that are located in the occipital horn of the LV, the posterior interhemispheric parieto-occipital transprecuneal trajectory provides an ideal corridor to achieve resection while minimizing the risks to the relevant subcortical tracts. For tumors that are isolated to the occipital horn and extend posteriorly or laterally toward the cortical surface, an occipital or posterior parietal transsulcal approach may be selected, depending on the superficial component of the tumor [7].

## Third ventricle

**Fig. 4:**
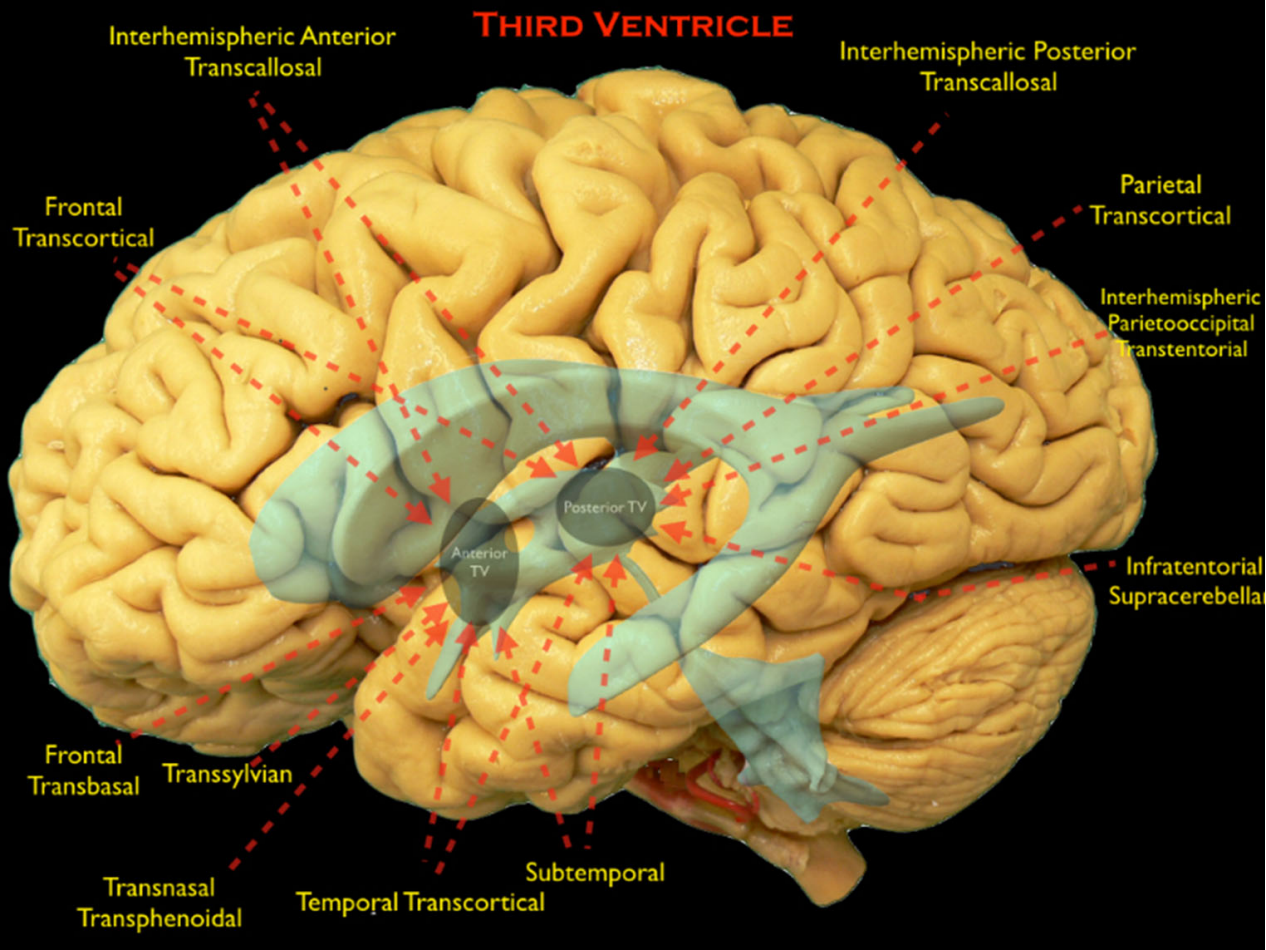
The surgical approaches to the third ventricle are shown on the lateral view of a cadaveric dissection of the brain. LV and TV are shown in *blue*. Parts of the TV are depicted with *gray circles*. *Red arrows* show the direction of the approaches and the parts of the TV that can be reached by that individual approach

**Fig. 5:**
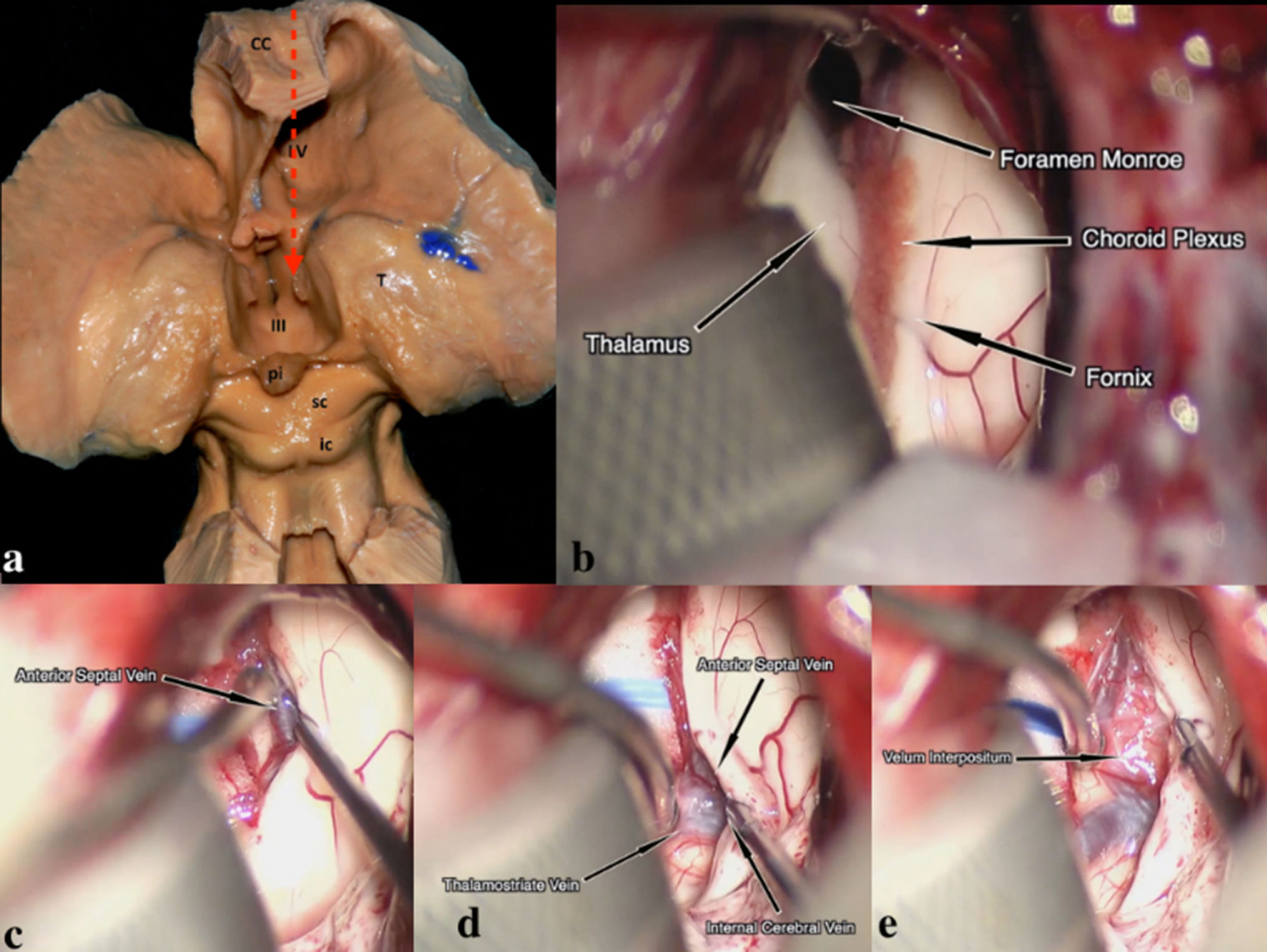
**a** The posterior part of the corpus callosum (CC) is removed, along with the posterior and superior walls of the LV, exposing the TV. The thalamus (T) forms the lateral walls of the posterior TV (III). The anatomical relation with the pineal gland (pi), superior colliculus (sc) and inferior colliculus (ic) can be seen. The *red arrow* shows the route leading to the FM and the TV through the CC. **b** An intra-operative picture demonstrating the anatomy of the choroidal fissure after entering to the LV. **c** Dissection between the fornix and the choroid plexus exposes the anterior septal vein. **d** The anterior septal vein and thalamostriate vein merge and form the internal cerebral vein. **e** Intraoperative picture revealing the velum interpositum (the roof of the TV) after retracting the venous structures and the choroid plexus

Tumors of the TV, just like the LV tumors, can be grouped into primary and secondary tumors. Primary tumors include colloid cysts, CP papillomas, ependymomas, subependymomas and central neurocytomas. The secondary group contains tumors such as craniopharyngiomas, pituitary tumors, hypothalamic gliomas, optic pathway gliomas, meningiomas and pineal region tumors [4, 24]. To gain access to these masses, surgeons must navigate around critical surrounding structures such as the hypothalamus, pituitary infundibulum, optic pathways, limbic system and their associated vascular structures [20, 42] (Figs. 4, 5). Possible complications include hemiparesis, seizures, visual loss, memory loss, and hypothalamic and pituitary dysfunction [24, 37, 40]. Poorly planned surgery may result in inadequate exposure, preventing gross total resection and risking significant neurological deficit.

### Open surgical approaches to the TV

Approaches to the TVF can be grouped into three broad categories: anterior, lateral and posterior routes. All of these approaches inevitably entail traversing unaffected neural tissues; therefore judicial selection of the operative route is especially important [38]. Tumor characteristics such as location, origin, extension, laterality, size, as well as the patient’s clinical status should be carefully considered in selection of the appropriate trajectory.

### Anterior approaches

After entering to LV via either IATcA or FTA, several routes can be used to reach the TV including transforaminal, interforniceal, transchoroidal and subchoroidal [39, 40]. The IATcA approach provides superior visualization of the entire cavity of the TV through multiple corridors. The distance to the TV via the IATcA is shorter than transcortical approach and is associated with a minimal risk of postoperative porencephaly, seizures and contralateral hemiparesis [41]. In the transforaminal approach, a natural orifice connecting the LVs and the TV, the foramen of Monroe (FM) is used to reach the anterior portion of the TV. This approach gives excellent exposure for small anterior TV tumors. Furthermore, larger tumors may be resectable via this approach if the tumor enlarges the FM. If necessary, this corridor can be extended either anteroposteriorly by cutting the ipsilateral fornix or posteriorly by dividing the thalamostriate vein [4, 42–44]. Sacrificing the fornix carries a significant risk of memory problems. Dividing the thalamostriate vein may result in drowsiness, hemiplegia, mutism, hemorrhagic infarct of the basal ganglia and even death [42] though some authors claim that unilateral thalamostriate vein sacrifice is well tolerated due to collateral circulation [43].

The choroidal fissure is a groove on the floor of the LV that is located between the fornix and the thalamus. The transchoroidal approach is based on dissection of the fissure to gain access to the roof of the TV and to its middle and posterior portions. After the dissection of the choroidal fissure is completed, the CP is retracted laterally to expose the velum interpositum (VIP), which forms the roof of the TV. Opening the VIP will create a corridor into the middle portion of the TV and it is even possible to reach tumors located in the posterior TV through this route. The internal cerebral vein (ICV), which lies within the VIP, must be preserved; injury to the ICV is one of the major risks of this approach [4, 45]. The surgical techniques for this approach are demonstrated in a video of the resection of a posterior TV ependymoma [Movie 4] (Fig. 3).

Retracting the CP medially and opening the corridor between the CP and the thalamus is known as the subchoroidal approach [45, 46]. Preserving the thalamostriate vein can be difficult with this approach, and it can be necessary for the vein to be coagulated and divided. The ICV is retracted medially with the CP. The VIP is then incised in the same manner as the transchoroidal approach [4, 20, 24, 43, 45–47]. The subchoroidal exposure carries the risk of injury to the thalamus, stria medullaris thalami, anterior and superior thalamic veins, thalamostriate vein, and the choroidal arteries [20, 24, 47]. With the subchoroidal approach, the fornix is well protected; however, this approach is used less frequently than the transchoroidal approach due to its increased risk of venous injury [42].

The interforniceal approach provides access to the anterior and central portions of the TV by dividing the midline forniceal raphe with subsequent opening of the roof of the TV along the plane between two forniceal bodies [3, 4, 38]. Unless there is midline shift, the septum pellucidum can be used as a guide. The presence of a cavum septum pellucidum is beneficial to minimize manipulation of the forniceal columns. This approach carries the risk of bilateral forniceal damage and subsequent profound memory problems [48]. The risk of bilateral forniceal damage has decreased the utilization of this approach. Exposure to the posterior TV is limited via this approach because the opening between the forniceal bodies should be limited to the anterior 1.5 cm to avoid damage to the forniceal commissure [11, 25]. Other important structures at risk during the interforniceal approach are ICVs and posterior medial choroidal arteries.

The subfrontal apprroach is useful for small anterior third ventricular tumors but provides limited access to the superior and posterior portions of the TV [37]. The subfrontal approach gives the best result for tumors involving the anteroinferior part of the TV that are not accessible via the transchoroidal approach [16, 49]. Several modifications of subfrontal approach are the translamina terminalis approach, the opticocarotid approach, the subchiasmatic approach, and the transnasal transsphenoidal approach [49–51].

The opticocarotid approach is the most useful for tumors extending superolaterally [20]. The position of the optic chiasm is divided into three: fixed, pre-fixed and post-fixed configurations. In the fixed and most common configuration, the optic chiasm is over the pituitary gland. A prefixed optic chiasm is located anteriorly over the tuberculum sella, whereas a post-fixed chiasm is located over the dorsum sella.

The subchiasmatic approach is advantageous when the optic chiasm is fixed or post-fixed. In patients with a prefixed chiasm, tumor resection is difficult, but if the lamina terminalis is stretched, the lamina terminalis approach may be beneficial. This approach provides adequate access to the anterior and inferior TV but has limited exposure of the FM or the roof [19, 37, 52]. Craniopharyngiomas are the most common tumors removed via this approach [20].

### Lateral approaches

The subtemporal approach is the main lateral corridor to the TV and is only recommended if the tumor is located lateral to the sella turcica or extends into the middle cranial fossa. Usually, the tumor mass is medial to the perforating branches of the posterior communicating artery, and it may be impossible to protect these vessels in some cases [20].

The pterional approach can also provide a narrow working channel toward the anterior TV after a wide dissection of the Sylvian fissure. Opening the lamina terminalis expands the exposure. This approach is commonly used for predominately third ventricular craniopharyngiomas. For multicompartmental tumors, the combination of this route with a transventricular approach (such as the interhemispheric transcallosal or transcortical transfrontal approach) is a valid option.

### Posterior approaches

The posterior wall of the TV is formed, in the rostral to caudal direction, by the splenium of the CC, the pineal gland and the tectum [49, 53, 54]. The supracerebellar infratentorial approach (ScItA), the interhemispheric posterior transcallosal approach (IPTcA), and the occipital transtentorial approach (OTtA) are commonly employed for tumors residing in the posterior TV [18, 25].

The ScItA is often used for tumors in the pineal region and posterior TV. The ability to visualize the tumors extending laterally and superiorly is limited during this approach [20]. The ScItA provides operative access to areas ranging from the transverse fissure of the cerebellum, quadrigeminal plate of the midbrain, the medial upper cerebellar peduncle, and the posterior TV [55]. The patient can be positioned either in a sitting or prone position. After the midline suboccipital craniotomy and dura opening, the bridging veins between the cerebellum and the tentorium, as well as the precentral cerebellar vein, can be sacrificed; however, the lateral dorsal cerebellar bridging veins and petrosal veins should be protected due to the risk of postoperative cerebellar venous congestion and swelling [56]. The arachnoid membranes overlying the pineal region are thickened, and careful dissection is required to avoid injury to the vein of Galen, basal veins of Rosenthal, and the ICVs. The natural corridor between the cerebellum and the tentorium provides straightforward access to the pineal region and posterior TV though the surgical corridor is relatively long and narrow [20, 54]. The slope of the tentorium narrows the operative field and restricts visualization both laterally and superiorly. Consequently, the ScItA is not suitable for tumors that extend rostrally above the tentorium or extend laterally into the atrium of the LV [38].

The IPTcA is similar to the IATcA except that the craniotomy is performed more posteriorly, and the callosotomy is conducted within the posterior aspect of the CC. The IPTcA is recommended for lesions in the posterior portion of the TV and the pineal region especially when there is a superior extension of the tumor involving the splenium of the CC [57]. The diencephalic veins are typically mobilized posteriorly. This approach can be performed with the patient in either the lateral or supine position. A parsagittal craniotomy that crosses to the contralateral side of the SSS is created, and the CC is exposed through the interhemispheric fissure. Consequently, this approach provides excellent visualization of the posterior TV and the pineal region; however, it fails to provide proper exposure of the lateral extent of the TV and carries the risk of damage to the deep venous system [58]. Transecting the posterior half of the CC can involve the posterior and habenular commissures, resulting in memory dysfunction and disconnection syndrome [20, 57, 59]. Use of this approach is limited to large tumors affecting the CC and splenium given the above mentioned complications of unaffected posterior callosotomy.

The OTtA is suitable for tumors in the pineal region extending into the posterior TV with a supratentorial component. A posterior callosotomy may not be necessary for this approach. This corridor is limited by poor visualization of the contralateral quadrigeminal region and ipsilateral pulvinar of the thalamus in the posterior TV [20]. Possible complications include damage to the midbrain and thalamus. [53]. Retraction of the occipital lobes should be avoided as this can lead to vision loss and care must be taken during incising the tentorium to avoid damage to the deep cerebral veins.

## Conclusions

Surgical excision is an important predictor of the outcome for tumors within the ventricular system. Origin, type, location and size of the tumor, age of the patient, patient co-morbidities, limitations in positioning, and tumor pathoanatomy should be carefully considered when choosing the appropriate approach for intraventricular tumors. Achieving a gross total resection of the tumor without significant complication requires a thorough understanding of available surgical approaches and their relative advantages and disadvantages.

## Electronic supplementary material

Below is the link to the electronic supplementary material.

Supplementary material 1 (MOV 281188 kb)

Supplementary material 2 (MP4 302950 kb)

Supplementary material 3 (MOV 172237 kb)

Supplementary material 4 (MP4 347440 kb)

## References

[1] Pendl G, Ozturk E, Haselsberger K (1992). Surgery of tumours of the lateral ventricle. Acta Neurochir (Wien).

[2] Delfini R, Acqui M, Oppido PA, Capone R, Santoro A, Ferrante L (1991). Tumors of the lateral ventricles. Neurosurg Rev.

[3] Vogel S, Meyer R, Lehmann R, Woiciechowsky C (1995). Transcallosal removal of lesions affecting the third ventricle:an anatomic and clinical study. J Neurosurg.

[4] Yasargil MG, Abdulrauf SI (2008). Surgery of intraventricular tumors. Neurosurgery.

[5] Lapras C, Deruty R, Bret PH, Symon L (1984). Tumours of the lateral ventricles. Advances and technical standards in neurosurgery.

[6] Gokalp HZ, Yuceer N, Arasil E, Deda H, Attar A, Erdogan A (1998). Tumors of the lateral ventricle: a retrospective review of 112 cases operated upon 1970–1997. Neurosurg Rev.

[7] Lucas TH, Ellenbogen RG (2001). Approaches to the ventricular system. Neurosurg Q.

[8] Ellenbogen RG (2001). Transcortical surgery for lateral ventricular tumors. Neurosurg Focus.

[9] Piepmeier JM (1996). Tumors and approaches to the lateral ventricles. J Neurooncol.

[10] Bernasconi V, Cabrini GP (1967). Radiological features of tumors of the lateral ventricles. Acta Neurochir (Wien).

[11] Fornari M, Savoiardo M, Morello G, Solero CL (1981). Meningiomas of the lateral ventricles. Neuroradiological and surgical considerations in 18 cases. J Neurosurg.

[12] Anderson RC, Ghatan S, Feldstein NA (2003). Surgical approaches to tumors of the lateral ventricle. Neurosurg Clin N Am.

[13] Yasargil MG (1996) Microneurosurgery: microneurosurgery of CNS tumors. Stuttgart, Georg Thieme Verlag, vol IVB, 38–42, 56–57, 63–65, 313–323

[14] Rhoton AL (2002). The lateral and third ventricles. Neurosurgery.

[15] Asgari S, Engelhorn T, Brondics A, Sandalcioglu IE, Stolke D (2003). Transcortical or transcallosal approach to ventricle-associated lesions: a clinical study on the prognostic role of surgical approach. Neurosurg Rev.

[16] Shucart WA, Stein BM (1978). Transcallosal approach to the anterior ventricular system. Neurosurgery.

[17] Izci Y, Seçkin H, Ateş O, Başkaya MK (2009). Supracerebellar transtentorial transcollateral sulcus approach to the atrium of the lateral ventricle: microsurgical anatomy and surgical technique in cadaveric dissections. Surg Neurol.

[18] Kawashima M, Li X, Rhoton AL, Ulm AJ, Oka H, Fujii K (2006). Surgical approaches to the atrium of the lateral ventricle: microsurgical anatomy. Surg Neurol.

[19] Abosch A, McDermott MW, Wilson CB, Kaye A, Black P (2000). Lateral ventricular tumors. Operative neurosurgery.

[20] Piepmeier JM, Westerveld M, Spencer DD, Sass KJ, Schmidek HH, Sweet WH (1995). Surgical management of intraventricular tumors of the lateral ventricles. Operative neurosurgicaltechniques: indications, methods, and results.

[21] D’Angelo VA, Galarza M, Catapano D, Monte V, Bisceglia M, Carosi I (2005). Lateral ventricle tumors: surgical strategies according to tumor origin and development—a series of 72 cases. Neurosurgery.

[22] Guidetti B, Delfini R, Gagliardi FM, Vagnozzi R (1985). Meningiomas of the lateral ventricles. Clinical, neuroradiologic, and surgical considerations in 19 cases. Surg Neurol.

[23] Heros RC (1990). Brain resection for exposure of deep extracerebral and paraventricular lesions. Surg Neurol.

[24] Strugar J, Piepmeier JM, Schmidek HH, Sweet WH (2000). Approaches to lateral and third ventricle tumors. Operative neurosurgical techniques: indications, methods, and results.

[25] Santoro A, Salvati M, Frati A, Polli FM, Delfini R, Cantore G (2002). Surgical approaches to tumours of the lateral ventricles in the dominant hemisphere. J Neurosurg Sci.

[26] Piepmeier JM, Spencer DD, Sass KJ, George TM, Apuzzo MLJ (1993). Lateral ventricular masses. Brain surgery: complication avoidance and management.

[27] Heilman KM, Gonzales Rothi LJ, Heilman K, Valenstein E (1985). Apraxia. Clinical neuropsychology.

[28] Diehl PR, Symon L (1981). Supratentorial intraventricular hemangioblastoma: case report and review of literature. Surg Neurol.

[29] Batjer H, Samson D (1987). Surgical approaches to trigonal arteriovenous malformations. J Neurosurg.

[30] Barrow DL, Dawson R (1994). Surgical management of arteriovenous malformations in the region of the ventricular trigone. Neurosurgery.

[31] Geffen G, Walsh A, Simpson D, Jeeves M (1980). Comparison of the effects of transcortical and transcallosal removal of intraventricular tumors. Brain.

[32] Ross ED, Kertesz A (1983). Right-hemisphere lesions in disorders of affective language. Localization in neuropsychology.

[33] Le Gars D, Lejeune JP, Peltier J (2009). Surgical anatomy and surgical approaches to the lateral ventricles. Adv Tech Stand Neurosurg.

[34] Shahinfar S, Johnson LN, Madsen RW (1994). Confrontation visual field loss as a function of decibel sensitivity loss on automated static perimetry. Ophtalmology.

[35] Yasargil MG, Türe U, Yasargil DC (2004). Impact of temporal lobe surgery. J Neurosurg.

[36] Yasargil MG, Wieser HG, Valavanis A, von Ammon K, Roth P (1993). Surgery and results of selective amygdala-hippocampectomy in one hundred patients with nonlesional limbic epilepsy. Neurosurg Clin N Am.

[37] Konovalov AN, Gorelyshev SK (1992). Surgical treatment of anterior third ventricle tumours. Acta Neurochir (Wien).

[38] Yamamoto I, Rhoton AL, Peace DA (1981). Microsurgery of the third ventricle: part I. Microsurgical anatomy. Neurosurgery.

[39] Timurkaynak E, Izci Y, Acar F (2006). Transcavum septum pellucidum interforniceal approach for the colloid cyst of the third ventricle operative nuance. Surg Neurol.

[40] Winkler PA, Ilmberger J, Krishnan KG, Reulen H-J (2000). Transcallosal interforniceal-transforaminal approach for removing lesions occupying the third ventricular space: clinical and neuropsychological results. Neurosurgery.

[41] Danaila L, Radoi M (2013). Surgery of tumors of the third ventricle region. Chirurgia.

[42] Türe U, Yaşargil MG, Al-Mefty O (1997). The transcallosal–transforaminal approach to the third ventricle with regard to the venous variations in this region. J Neurosurg.

[43] Lavyne MH, Patterson RH (1983). Subchoroidal trans-velum interpositum approach to mid-third ventricular tumors. Neurosurgery.

[44] Yasargil MG, Curcic M, Kis M, Siegenthaler G, Teddy PJ, Roth P (1990). Total removal of cranio-pharyngiomas: approaches and long-term results in 144 patients. J Neurosurg.

[45] Cossu M, Lubinu F, Orunesu G, Pau A, Sehrbundt Viale E, Sini MG (1984). Subchoroidal approach to the third ventricle. Microsurgical anatomy. Surg Neurol.

[46] Yilmaz T, Cikla U, Başkaya MK (2015) Microsurgical treatment of thalamic cavernous malformation: 3-Dimensional operative video. Neurosurgery. [Epub ahead of print] PMID:26308627 10.1227/NEU.0000000000000964 29506173

[47] Rhoton AL, Yamamato I, Peace DA (1981). Microsurgery of the third ventricle: part 2. Neurosurgery.

[48] Hassaneen W, Suki D, Salaskar AL, Levine NB (2010). Immediate morbidity and mortality associated with transcallosal resection of tumors of the third ventricle. J Clin Neurosci.

[49] Herrmann HD, Winkler D, Westphal M (1992). Treatment of tumours of the pineal region and posterior part of the third ventricle. Acta Neurochir (Wien).

[50] Kulwin C, Chan D, Ting J, Hattab EM, Cohen-Gadol AA (2014). Endoscopic endonasal transplanum transtuberculum resection of a large solid choroid plexus papilloma of the third ventricle. J Clin Neurosci.

[51] Johnson RR, Baehring J, Piepmeier J (2003). Surgery for third ventricular tumors. Neurosurg Q.

[52] Oi S, Samii A, Samii M (2003). Operative techniques for tumors in the third ventricle. Op Tech Neurosurg.

[53] Fukui M, Natori Y, Matsushima T, Nishio S, Ikezaki K (1998). Operative approaches to the pineal region tumors. Child’s Nerv Syst.

[54] Lozier AP, Bruce JN (2003). Surgical approaches to posterior third ventricular tumors. Neurosurg Clin N Am.

[55] Laborde G, Gilsbach JM, Harders A, Seeger W (1992). Experience with the Infratentorial supracerebellar approach in lesions of the quadrigeminal region, posterior third ventricle, culmen cerebelli, and cerebellar peduncle. Acta Neurochir (Wien).

[56] Little KM, Friedman AH, Fukushima T (2001). Surgical approaches to pineal region tumors. J Neuro Oncol.

[57] Schijman E (1989). Microsurgical anatomy of the transcallosal approach to the ventricular system, pineal region and basal ganglia. Child’s Nerv Syst.

[58] Benes V (1990). Advantages and disadvantages of the transcallosal approach to the III ventricle. Child’s Nerv Syst.

[59] Jia W, Ma Z, Liu IY, Zhang Y, Jia G, Wan W (2011). Transcallosal interforniceal approach to pineal region tumors in 150 children. J Neurosurg Pediatr.

